# Alcohol as a Disinfectant

**Published:** 1898-04

**Authors:** 


### Alcohol as a Disinfectant.

Recent researches seem to show that absolute alcohol is devoid of all disinfectant properties, whereas proof spirit (50 percent) gives more tangible results in this direction than either stronger or weaker solutions. Antiseptic substances which, in aqueous solution, are more or less active germicides, entirely lose this property when dissolved in strong alcohol, but, on the other hand, corrosive sublimate, carbolic acid, lysol, and thymol, dissolved in a 50 percent solution of alcohol, disinfect better than aqueous solutions of the same strength.—*Med. Press and Circular.*

